# Supplementary material for: Collaborative intelligence in AI: Evaluating the performance of a council of AIs on the USMLE
Source: PLOS Digit Health. 2025 Oct 9;4(10):e0000787. doi: 10.1371/journal.pdig.0000787 (PMC12510544; doi:10.1371/journal.pdig.0000787)
Supplement: S2 File — (ZIP) [file pdig.0000787.s002.zip › S2 Data Raw data files containing unprocessed question inputs and LLM outputs including transcripts of deliberations/STEP3_Question-64-1_Type-MC-NJ.docx]

**Facilitator** **[Round 1]:** A 45-year-old woman with a history of seizure disorder comes to the office because she has had seizures daily for the past 3 weeks, despite adhering to her medication regimen. Medical history is also significant for borderline hypertension for the past year and alcohol use disorder. She admits that she has been drinking two beers daily since she lost her job 3 weeks ago. Medications include phenobarbital. Family history is significant for type 2 diabetes mellitus and hypertension in both parents and in all of her grandparents, heart disease in her sister, and death of her paternal grandfather of complications from alcohol use disorder. The patient smoked one pack of cigarettes daily for 20 years but quit smoking 1 year ago. Since losing her job, the patient has been earning money by driving her neighbors' children to school and driving another neighbor to hospital appointments. BMI is 22 kg/m². Vital signs are temperature 37.1°C (98.8°F), pulse 80/min, respirations 16/min, and blood pressure 138/86 mm Hg. Physical examination discloses no abnormalities. Serum phenobarbital concentration is obtained and results are pending. Which of the following is the priority in management at today's visit? (A) Discussing a new treatment regimen for the patient's seizure disorders (B) Evaluating the patient for depressive disorder (C) Evaluating the patient's ability/desire to stop drinking alcoholic beverages (D) Instructing the patient to stop driving

**AI_C:** RESTATING THE QUESTION: The question wants to identify the most imperative action that needs to be taken in the management of a 45-year-old woman that is dealing with an increased frequency of seizures despite her medication regimen. Her history comprehends borderline hypertension, alcohol use disorder, and a significant familial predisposition for a set of various diseases. She is currently on phenobarbital for her seizure disorder. Since losing her job 3 weeks ago, she has increased her daily alcohol consumption and started driving children to school and another neighbor to hospital appointments to earn money. PLANNING: The assumptions that could be made from the information presented in the question include a potential effect of alcohol on the seizure medication's effectiveness, a possible role of stress and depression in exacerbating her condition following her job loss, a potential symptom of a more serious underlying condition, or the implications of her action of driving while suffering regular seizures. It is also crucial to assess the relationship between these assumptions, her history of alcohol use, job loss, and the exacerbation of her seizures. It's vital to prioritize the implication that poses the greatest risk to her and those around her, which in this case is the possibility of an accident occurring due to a seizure while she is driving. CLINICAL REASONING: Her history of regular seizures, despite medication adherence, combined with her current livelihood, which involves driving neighbors to their appointments, encompasses a major safety concern not only for her, but also for others on the road. Lack of control over seizures greatly increases the risk of accidents while driving and should be addressed urgently in her management. In seizure disorders, it is the frequency and unpredictability of the seizure episodes that determines the driving restrictions. While it is also important to examine her increased alcohol intake and evaluate the interaction with her current medication, also considering adjusting her treatment plan or evaluating for depressive disorder, these actions do not deal with the immediate potential dangers associated with uncontrolled seizures and driving. CONCLUDING: In consideration of the immediate and potential risk imposed by the patient's current condition and lifestyle activities, priority should be focused on safety, which means invoking restrictions on her driving until her seizures are better controlled. The other considerations (alcohol consumption, depression, treatment adjustment) remain important and should be pursued subsequently, but the driving issue constitutes an urgent public health matter. RESPONSE: The priority in management at today's visit is instructing the patient to stop driving (Option D).

**AI_D:** RESTATING THE QUESTION: A 45-year-old female who has been suffering from seizure disorder is visiting the office as her seizures have been occurring daily for the past three weeks. This is happening regardless of her compliance with her medication regimen that includes phenobarbital. Her health history also reveals borderline hypertension for the past year and alcohol use disorder. She has increased her alcohol consumption to two beers daily subsequent to losing her job about three weeks ago. Her family history is marked with type 2 diabetes and hypertension in all parents and grandparents, heart disease in her sister, and mortality of her paternal grandfather owing to alcohol use disorder. While she quit smoking a year ago after twenty years of daily smoking, she has taken up driving jobs for income since her job loss. Her vital signs seem normal with a slight hypertension. The concentration of phenobarbital in her serum is under analysis. The primary concern to address in her management today is being determined from the following: a new regimen for her seizures, evaluation for depressive disorder, exploration of her ability and willingness to quit alcohol, and urging her to cease driving. PLANNING: The primary assumption seems to be that increased seizure frequency is due to inadequate control from the current regimen. However, other possibilities involve interaction of phenobarbital with alcohol, effect of stress (due to job loss) on seizure control, or alcohol withdrawal seizures. Evaluating her psychological health status is also an important area to consider. Another critical aspect is her safety, given she is driving regularly despite increased seizures. CLINICAL REASONING: The fact that the seizures increased in frequency following her job loss and increased alcohol consumption suggests that these factors could be influencing her seizure control; either due to alcohol's interaction with phenobarbital or due to withdrawal seizures. Stress due to job loss could be another contributing factor. In the instance of this patient, considering depressive disorder might also be pertinent given her job loss, increased alcohol intake, and daily seizures, which could lead to feelings of hopelessness or depression. However, an immediate threat to her safety is her continued driving given her daily seizures, as this could result in a serious accident threatening her life and potentially others'. CONCLUDING: While other medical issues should also be addressed, the immediate priority should be ensuring her safety, which is compromised by her continued driving despite her increased seizure frequency. Inappropriate seizure control can be addressed simultaneously with a modification of her medication, but the immediate life-threatening situation results from her continuing to drive. The ability to evaluate her mental health status and manage her alcohol consumption is also paramount, but safety should be addressed first. RESPONSE: Instructing the patient to stop driving (Option D) should be the priority in management at today's visit. Ensuring the safety of the patient is a primary concern in healthcare. Additionally, her being a potential risk to others on the road makes this an urgent priority. Following this, addressing her inadequate seizure control, possible depression and alcohol use disorder can be initiated.

**AI_B:** RESTATING THE QUESTION: A 45-year-old woman comes to the clinic with an increase in the frequency of her seizures. These are occurring daily and have been happening for the past three weeks. This increase is despite her faithfully taking her anti-seizure medication, phenobarbital. Her other history is remarkable for borderline hypertension, alcohol use disorder, and smoking for 20 years although she quit last year. Currently, she has been consuming two beers daily for three weeks since she lost her job. She is driving children and a neighbour as a way of generating income. Her vitals are within range, but her blood pressure is at the upper limit. There are no physical abnormalities, and her serum phenobarbital concentration is being evaluated. The best course of action for her treatment in today's visit needs to be identified. PLANNING: The main assumptions to consider are that her seizures are due to her existing seizure disorder, the increase may be due to her continuous alcohol intake or due to an ineffective treatment regimen, her depression, or her ability to safely drive. By evaluating her medical and family history, mental health, lifestyle, and habits, the priority can be ascertained. CLINICAL REASONING: Seizures occur when there's a sudden surge of electrical activity in the brain. This woman has seen an increase in seizure frequency despite her regimen. Alcohol withdrawal can induce seizures; she's increased her intake recently and although she's not stopped drinking, it's possible she isn't maintaining a consistent blood alcohol level. Phenobarbital is a long-acting barbiturate and anticonvulsant used in managing seizures. Its effectiveness can be compromised by alcohol which is a central nervous system suppressant, probably resulting in subtherapeutic levels despite correct dosage. This is also supported by her clinical presentation of no abnormal physical findings, which points towards a biochemical cause. While it is essential to discuss the possible ineffectiveness of the current regimen, the priority should be to address the potential imminent risk, which is the interaction of alcohol with phenobarbital causing an uptick in seizures. CONCLUDING: Alcohol has severe interactive effects with phenobarbital which can cause subtherapeutic serum levels thus not adequately controlling her seizures. Continued drinking and driving post-seizure also puts her and others at risk. RESPONSE: Therefore, the priority in her management at today's visit should be (C) Evaluating the patient's ability/desire to stop drinking alcoholic beverages. It is crucial to discuss the interaction between her medication and alcohol while initiating necessary steps for managing her alcohol use disorder.

**AI_E:** RESTATING THE QUESTION: The query is about prioritizing the management for a 45-year-old woman. She has a background of seizure disorder and has been experiencing daily seizures for three weeks despite her medication. Her medical history also includes borderline hypertension and alcohol use disorder. The patient has been drinking two beers daily after losing her job three weeks ago. Her medication includes phenobarbital. The patient's family history is notable for type 2 diabetes, hypertension, heart disease, and complications from alcohol use disorder. The patient formerly had a smoking habit and has quit since 1 year, and has assumed the role of a driver after losing her job. Her vital signs and physical examination are within normal limits. A phenobarbital concentration has been obtained and is currently under study. Now, the query is seeking to identify the top priority in managing her health. PLANNING: A few assumptions can be made based on the information given. 1) The patient's increased seizure activity could be due to inadequate control from her current medication, phenobarbital. 2) Her job loss and increased alcohol intake might have contributed to a depressive disorder. 3) The patient's increased alcohol intake could be an attempt to cope with her job loss and it might exacerbate her existing health conditions. 4) Her role as a driver could be dangerous considering her daily seizures. After generating these assumptions, it appears that her uncontrolled seizures and her duty as a driver pose immediate health and safety threats, respectively. Therefore, assessing the priority between these two becomes clinically relevant. CLINICAL REASONING: Given her daily seizures, this surely could impair her ability to drive, posing a safety risk to both the patient and the individuals she is driving. The introduction of a new treatment regimen or her underlying depressive disorder, or her desire to quit drinking does not present as immediate of a risk to her and other individual's safety. Furthermore, seizures can lead to potential trauma and sudden loss of consciousness while driving could potentially lead to fatal accidents. CONCLUDING: Given that the life-threatening risk of continuing to drive with her current level of seizure control outweighs the other potential management strategies, addressing this as a priority is significant. The other aspects such as adjusting her treatment regimen for seizures, potential for depression and her alcohol consumption problems can be addressed once this immediate risk has been managed. RESPONSE: Therefore, the priority in managing the patient at today's visit (D) Instructing the patient to stop driving. Her uncontrolled seizures make it unsafe for her to be driving, and addressing this is the most urgent concern. Afterward, the other aspects of her health condition should be addressed.

**AI_A:** RESTATING THE QUESTION: Essentially, we are being asked how we should prioritize the management of a woman with seizure disorder, who has been having more frequent seizures in the past few weeks in spite of medication. Her medical history also includes borderline hypertension, alcohol use disorder, and she indulges in two beers daily since losing her job three weeks ago. Her treatment includes phenobarbital. Other crucial information includes her family's medical history, her previous smoking habit, her current job as a driver, and her medically stable condition, except for slightly high blood pressure. The clinician is now considering what should be the immediate clinical priority at this visit. PLANNING: There are several interlinked factors that could potentially be causing her seizures: (a) her alcohol use disorder and her recent increment in alcohol consumption could potentially interact with her phenobarbital and lead to insufficient blood levels of the drug; (b) the stress from her job loss could potentially exacerbate her seizures; (c) her hypertension might be contributing to her seizure disorder; and (d) her current job as a driver could pose significant risks given her daily seizures. It is crucial to assess and verify if her phenobarbital levels are within the therapeutic range, whether she is willing to stop/reduce her alcohol consumption, if she is experiencing depression due to her job loss, or if she understands the danger of driving with uncontrolled seizures. CLINICAL REASONING: From the given details, we can deduce that the woman's recent uptick in seizure frequency coincides with her job loss and consequent increase in daily alcohol consumption. Phenobarbital, her antiepileptic medication, is a barbiturate that can be influenced by alcohol, which also has CNS depressive properties. This interaction may lower the plasma levels of phenobarbital and hence contributed to her poor seizure control. As a result, it is plausible to think that addressing her alcohol consumption may improve her seizure management. Additionally, given that she is ferrying children and adults to school and hospital appointments respectively, her uncontrolled seizures have significant safety implications. CONCLUDING: In light of the aforementioned sequencing of events and potential impacts, her drinking situation appears to be the immediate factor impacting her seizure control and real-time safety. Therefore, it becomes a priority before adjusting her seizure medication regimen or investigating for depression. RESPONSE: Therefore, the appropriate approach to this patient's management during this visit would be to evaluate the patient's ability/desire to stop drinking alcoholic beverages (Option C). However, it is also simultaneously crucial for the patient to stop driving until her seizures are well controlled, for safety reasons.

**Facilitator** **[Round 2]:** Summary: AI_A and AI_B both believe that the immediate priority should be to evaluate the patient's ability/desire to stop drinking alcoholic beverages. They argue that the woman's increased alcohol consumption could be interacting with her phenobarbital medication, leading to insufficient blood levels of the drug and hence poor seizure control. They also note the safety implications of her driving with uncontrolled seizures, but see this as a secondary concern to be addressed after her alcohol consumption. AI_C, AI_D, and AI_E, on the other hand, believe that the immediate priority should be to instruct the patient to stop driving. They argue that her daily seizures pose a significant safety risk to both herself and others on the road. While they acknowledge the potential impact of her increased alcohol consumption on her seizure control, they see this as a secondary concern to be addressed after her driving. Key Question: To clarify the disagreements between their opinions, the council members could be asked: "Given the potential for both the patient's increased alcohol consumption and her driving to pose significant safety risks, how should these risks be weighed against each other in determining the immediate clinical priority?". Based on your preceding response, how would you answer the original question: [A 45-year-old woman with a history of seizure disorder comes to the office because she has had seizures daily for the past 3 weeks, despite adhering to her medication regimen. Medical history is also significant for borderline hypertension for the past year and alcohol use disorder. She admits that she has been drinking two beers daily since she lost her job 3 weeks ago. Medications include phenobarbital. Family history is significant for type 2 diabetes mellitus and hypertension in both parents and in all of her grandparents, heart disease in her sister, and death of her paternal grandfather of complications from alcohol use disorder. The patient smoked one pack of cigarettes daily for 20 years but quit smoking 1 year ago. Since losing her job, the patient has been earning money by driving her neighbors' children to school and driving another neighbor to hospital appointments. BMI is 22 kg/m². Vital signs are temperature 37.1°C (98.8°F), pulse 80/min, respirations 16/min, and blood pressure 138/86 mm Hg. Physical examination discloses no abnormalities. Serum phenobarbital concentration is obtained and results are pending. Which of the following is the priority in management at today's visit? (A) Discussing a new treatment regimen for the patient's seizure disorders (B) Evaluating the patient for depressive disorder (C) Evaluating the patient's ability/desire to stop drinking alcoholic beverages (D) Instructing the patient to stop driving]

**AI_C:** RESTATING THE QUESTION: The presented dilemma underlines the need to prioritize the medical and personal aspects of the case of a 45-year-old woman who has been having seizures daily. These seizures remain uncontrolled despite her sticking to her prescribed medication regimen. Alcohol usage disorder is also a pivot in her case, with her recent increase in alcohol consumption. She has become a part-time driver since her job loss, whilst her seizures continue unabated. PLANNING: We can identify several assumptions, the first being her uncontrolled seizures due to below-optimal phenobarbital dosage exacerbated by alcohol consumption. Another assumption could be the risk she poses to public safety by driving uncontrolled seizures. The presence of depressive disorder can't be disregarded considering her job loss, daily alcohol use, and the associated stigma of her illness. Clinical prioritization nuances dictate immediate addressing of risks to patient life or safety. CLINICAL REASONING: Looking at these assumed clinical scenarios, the most pressing clinical issue appears to be the patient's uncontrolled seizures and her adherence to driving. Seizures coupled with driving may lead to accidents resulting in severe injury or fatality to her and others in the vicinity. This can compounded by her elevated alcohol intake which can also interfere with the phenobarbital's efficacy. CONCLUDING: On the balance of probabilities, the risk of severe injury or fatality due to a seizure while driving is an immediate threat to her and public safety which outweighs other potential underlying conditions such as the possibility of her depressive disorder or addiction. RESPONSE: Prioritizing safety for both the patient and the public leads me to agree that her immediate priority should be that she suspends her driving activities until the seizures are controlled, thus, the option (D) Instructing the patient to stop driving would be the appropriate answer.

**AI_A:** Restating the question, we need to prioritize the management approach for a 45-year-old woman who has been experiencing uncontrolled seizures in spite of medication adherence, has a history of borderline hypertension, and disclosed increased alcohol consumption since she lost her job 3 weeks ago. Phenobarbital is her medication, and she has significant family history for type 2 diabetes mellitus, hypertension, heart disease, and alcohol use disorder. She has also assumed the responsibility of driving since losing her job, even with her ongoing seizures. These duties include driving neighborhood children to school and ferrying another neighbor to hospital appointments. For planning, several underlying assumptions can be identified. Firstly, the woman's increased alcohol consumption could be interfering with her medication therapy by lowering the blood serum concentration of phenobarbital, resulting in daily seizures. This assumption should be clinically prioritized; alcohol use disorder can be quite harmful to a patient on phenobarbital due to the potential of drug-alcohol interactions leading to reduced therapeutic efficacy of her medication (phenobarbital). For clinical reasoning, phenobarbital is a medication that treats seizure disorders. However, alcohol can interact with the drug, either by affecting its metabolism, hence its efficacy, or by heightening the sedative effects of phenobarbital. This could put the patient in danger, particularly as she drives neighborhood children to school and a neighbor to hospital appointments with uncontrolled seizures. In conclusion, it is paramount to clarify and address her alcohol consumption as it directly relates to her seizure medication and control of the condition. The fact that this has a potential bearing on her medication effectiveness and her current daily seizures makes it a priority. This is not to undermine the immediate risk she presents when she drives with uncontrolled seizures. However, addressing alcohol intake will also indirectly affect this risk as controlling her seizures will improve her safety while driving. Thus, my response will be option (C) Evaluating the patient's ability/desire to stop drinking alcoholic beverages. This should be considered as the priority management strategy at this visit due to the possibility of an interaction between her increased alcohol intake and her phenobarbital medication and its implications on her seizures. The wellbeing of the patient and those she interacts with while driving is paramount–it is a matter of immediate health and public safety. Consequently, her ability or desire to cease drinking is a crucial point of intervention to discuss.

**AI_B:** RESTATING THE QUESTION: This scenario involves a 45-year-old woman who is experiencing an increased frequency of seizures despite being on phenobarbital. She also has other health issues such as borderline hypertension and alcohol use disorder. Since losing her job, she has increased her alcohol consumption and is now driving as a source of income. We are asked to determine the priority in this patient's management. PLANNING: There are several assumptions made in each management option. For (A), it assumes her seizures are not adequately controlled by her current medication. For (B), it assumes she may have depressive disorder due to her recent job loss and increased alcohol use. For (C), it assumes that her alcohol use could be affecting her seizure control or overall health. For (D), it assumes that her seizures pose a potential threat to road safety. Each assumption can be questioned and have implications on her overall health and safety. However, the clinically prioritized assumption that directly impacts her and other individuals’ immediate safety would be her driving with uncontrolled seizures. CLINICAL REASONING: To evaluate the priority assumption, a clinical understanding of the pathology and pharmacology of her situation is crucial. Alcohol is known to decrease the effectiveness of phenobarbital, reducing its seizure control capabilities. However, it is also true that uncontrolled seizures can impair her driving, posing a threat to her and others. While addressing her alcohol consumption is crucial for long-term management, the immediate risk of harm from uncontrolled seizures while driving should be addressed first. CONCLUDING: Given the patient's present condition, her driving habits present an immediate safety risk not only to herself but to others on the road as well. Even if her alcohol use disorder could potentially be impacting her seizure control, the current driving practice remains an immediate danger that should be addressed promptly. RESPONSE: Therefore, based on the above analysis, the priority in management at today's visit should be (D) Instructing the patient to stop driving. This is because the safety implications of uncontrolled seizures while driving are immediate and severe. This does not negate the need to address her alcohol use and the efficacy of her medication, but it does prioritize the immediate danger on the road. Therefore, usually these situations should be managed with a multi-disciplinary approach later on to ensure her health condition is completely treated.

**AI_D:** Restating the question, we have to determine the immediate priority in managing a 45-year-old woman presenting with daily seizures for the past three weeks, despite adherence to her medication regimen with phenobarbital. She also reports increased daily alcohol consumption and has a significant risk factor profile like a family history of hypertension, type 2 diabetes mellitus, and alcohol use disorder. To plan, we should note that phenobarbital is a sedative medication used to treat seizures. However, it can be significantly impacted by alcohol consumption, which can lower its effectiveness and increase the risk for uncontrolled seizures. Furthermore, the woman is currently driving regularly, which poses a significant safety risk for herself and others given her daily seizures. There are multiple assumptions here, the first being that the woman's increased alcohol consumption is the primary cause of her poor seizure control. An alternate assumption could be that her phenobarbital medication regimen is inadequate for her seizure disorder. Still, we also have an immediate safety concern regarding her continuous driving despite daily seizures. Given the potentially severe outcomes associated with uncontrolled seizures while driving, this issue takes precedence in the decision-making process. In the clinical reasoning process, it's crucial to address both the immediate safety concern of the patient's driving and her persistent, uncontrolled seizures despite medication, which could be a result of her alcohol consumption. However, since driving with uncontrolled seizures poses an immediate risk to both the patient and the public, this concern should be addressed first before focusing on her seizure control and alcohol consumption. Concluding, it is imperative to consider immediate safety risks when determining the primary management priority in patient care. In this case, the woman's continued driving despite daily seizures represents such a risk, outweighing the importance of immediately addressing her increased alcohol consumption or her inadequate seizure control. Therefore, in response to the original question, the priority in management at today's visit would be (D) Instructing the patient to stop driving due to the considerable risk it poses to her and the public. After which, reevaluating her treatment regimen alongside her alcohol use disorder would follow as important considerations.

**AI_E:** RESTATING THE QUESTION: We need to determine the clinical priority in the management of a patient who has been experiencing daily seizures despite compliance with her medication regimen. She has a history of seizure disorder, borderline hypertension, and alcohol use disorder. She has been consuming two beers daily since she lost her job three weeks ago. She has also been driving children to school and a neighbor to hospital appointments as a way to earn money. Her medication includes phenobarbital. Her health and family history, as well as her current lifestyle and behavior, need to be taken into consideration. PLANNING: In managing this patient, we have to make an assumption. One might assume that her increased alcohol consumption is interacting with her phenobarbital, leading to ineffective blood levels of the drug, and hence, poor seizure control (assumption 1). Another assumption would be that her seizure disorder makes her current activities, such as driving children to school and a neighbor to hospital appointments, a significant safety risk to both herself and others on the road (assumption 2). Given the severity of the potential repercussions of both scenarios, it is important to prioritize which assumption to pursue first. CLINICAL REASONING: Phenobarbital, a medication the patient is currently taking, is a long-acting barbiturate generally used in the treatment of seizures. It can be affected by chronic alcohol consumption, as both substances induce the hepatic cytochrome P450 enzyme system, leading to the increased metabolism of phenobarbital and, consequently, decreased drug levels in the blood (supporting assumption 1). Reduced levels of phenobarbital could lead to uncontrolled seizures. Moreover, this daily alcohol consumption poses a direct threat to her health, and possibly to her seizure control. On the other hand, her current job of driving, along with her daily seizures, undeniably pose a substantial risk for accidents (providing support to assumption 2). Not only would an accident potentially be dangerous or fatal for her, but it could also harm others, especially the children she is driving to school and the neighbor she is driving to the hospital. CONCLUDING: Safety—both the patient's and the public's—should remain the top priority in healthcare. Between the two assumptions, the urge to address her daily alcohol consumption because it could be influencing the therapeutic level of her seizure medication given the increased risk of harm to multiple parties from her current daily activity, namely driving, should be given precedence (pointing to assumption 2). RESPONSE: Therefore, the priority in managing this patient at today's visit should be D) Instructing the patient to stop driving. Once the immediate risk of harm to herself and others is addressed, next steps would involve exploring her alcohol use and its potential impact on her seizure control, as well as the need for potential adjustment of her treatment regimen.

**Facilitator** **[Round 3]:** Summary: AI_A believes that the priority should be evaluating the patient's ability/desire to stop drinking alcoholic beverages. They argue that the woman's increased alcohol consumption could be interfering with her medication therapy, leading to uncontrolled seizures, especially dangerous given her driving responsibilities. AI_B, AI_C, AI_D, and AI_E all agree that the immediate priority should be instructing the patient to stop driving. They argue that the immediate risk of harm from uncontrolled seizures while driving should be addressed first, even though addressing her alcohol consumption is crucial for long-term management. Key Differences: The key difference in the perspectives lies in the immediate priority for the patient's management. AI_A prioritizes addressing the patient's alcohol consumption due to its potential interaction with her medication and its implications on her seizures. In contrast, AI_B, AI_C, AI_D, and AI_E prioritize addressing the immediate danger of the patient driving with uncontrolled seizures, considering it a direct threat to her and public safety. Question for Clarification: Given the potential for both alcohol consumption and uncontrolled seizures to pose significant risks to the patient and public safety, how would you rank these risks in terms of immediacy and severity? Would your ranking change if the patient's driving responsibilities were removed from the equation?. Based on your preceding response, how would you answer the original question: [A 45-year-old woman with a history of seizure disorder comes to the office because she has had seizures daily for the past 3 weeks, despite adhering to her medication regimen. Medical history is also significant for borderline hypertension for the past year and alcohol use disorder. She admits that she has been drinking two beers daily since she lost her job 3 weeks ago. Medications include phenobarbital. Family history is significant for type 2 diabetes mellitus and hypertension in both parents and in all of her grandparents, heart disease in her sister, and death of her paternal grandfather of complications from alcohol use disorder. The patient smoked one pack of cigarettes daily for 20 years but quit smoking 1 year ago. Since losing her job, the patient has been earning money by driving her neighbors' children to school and driving another neighbor to hospital appointments. BMI is 22 kg/m². Vital signs are temperature 37.1°C (98.8°F), pulse 80/min, respirations 16/min, and blood pressure 138/86 mm Hg. Physical examination discloses no abnormalities. Serum phenobarbital concentration is obtained and results are pending. Which of the following is the priority in management at today's visit? (A) Discussing a new treatment regimen for the patient's seizure disorders (B) Evaluating the patient for depressive disorder (C) Evaluating the patient's ability/desire to stop drinking alcoholic beverages (D) Instructing the patient to stop driving]

**AI_B:** Understanding the question: This scenario presents a complex case of a 45-year-old woman with a history of seizure disorders who has been experiencing daily seizures for the past three weeks, despite her medication regimen. She also has a history of borderline hypertension, alcohol use disorder, and has a family history of several serious ailments. She has recently lost her job and has been drinking beer daily, which could be interfering with her medication and exacerbating her seizures. The crux of this scenario, however, is that the patient has been driving people in her neighborhood to earn money, thus potentially posing a significant risk to the public, particularly because of her uncontrolled seizures. Planning: Two primary assumptions emerge from this case: the woman's increased alcohol consumption is interfering with her seizure medication, and her uncontrolled seizures pose immediate danger when she's driving. Alternate assumptions could include that her medication regimen needs adjustment or she is suffering from a depressive disorder following the loss of her job. Yet, the scenarios with more immediate life-threatening risks are her propensity to drink while on medication and her driving despite uncontrolled seizures. Clinical Reasoning: Both emphasized hypotheses could be directly causing her seizures to remain uncontrolled. Alcohol consumption can interfere with phenobarbital's therapeutic levels by inducing the enzymes responsible for drug metabolism, which ultimately reduces the drug's ability in maintaining seizure control. On the other hand, having a seizure while driving could potentially lead to a fatal accident. Concluding: Prioritizing the urgency of these hypotheses depends on the immediate threat they pose. While the daily consumption of alcohol indeed poses significant risks to managing her underlying health conditions in the long term, the immediacy and gravity of harm caused by a potential motor vehicle accident due to an uncontrolled seizure should be clinically prioritized. Response: Thus, given this context, the priority should be instructing the patient to stop driving (D). This action will address an immediate risk to both the patient's and public safety. However, it should also be emphasized that this does not eliminate the immediate need to address the alcohol use disorder which might be possibly complicating her medication management and seizure control.

**AI_C:** Given the scenario, the patient is facing a multifaceted problem, ranging from uncontrolled seizures to an increase in alcohol consumption, heightened by her daily driving duties. Restating the Question: The patient has uncontrolled seizures despite medication adherence and has been driving regularly while dealing with this condition. Alcohol consumption and hypertension are also factors. We are asked to identify the immediate management priority. The choices are a new treatment for seizures, evaluation for depressive disorder, assessment of alcohol cessation, and instruction to stop driving. Planning: Several assumptions emerge. Firstly, it's presumed that the woman's alcohol use or stress from job loss may be exacerbating her seizures. Secondly, it's assumed that uncontrolled seizures make driving unsafe. Thirdly, the presence of hypertension and a family history of cardiovascular disorders warrants consideration. Clinical reasoning: Phenobarbital, her antiepileptic drug, is a moderate inducer of the cytochrome P450 system, which metabolizes many substances, including alcohol. Her increased alcohol consumption could alter the drug's metabolism, causing ineffective seizure control. Concurrently, regular drinking may exacerbate her borderline hypertension. However, these are long-term issues that will, undoubtedly, need addressing but do not require immediate intervention. The critical issue here is her uncontrolled seizures, which, coupled with her daily driving, pose a significant immediate risk to her and public safety. Concluding: The main concern is the immediate and severe risk posed by uncontrolled seizures while driving. Accordingly, the priority is to instruct the patient to stop driving until her seizures are under control. Regardless of her financial situation or reliance on driving as a source of income, her safety and the safety of others is paramount. Response: Therefore, the answer would be (D) Instructing the patient to stop driving. Although other areas like her alcohol consumption, need to be addressed in the long-term management of her health, the immediate priority is to manage the acute risk posed by her uncontrolled seizures while driving.

**AI_D:** Restating the question: The question presented is asking us to identify the most pressing concern in the management of a 45-year-old woman who is experiencing daily seizures despite medication and who has recently increased her alcohol consumption following job loss. She is also driving other people's children to school, exposing them to elevated risk from her seizure disorder. Planning: The key assumption is that because she is having daily seizures despite being on medication and drinking alcohol, her alcohol use might be affecting the effectiveness of her medication, or her seizures might be worsening due to stress or other reasons related to job loss. That's why one school of thought is to evaluate her ability/desire to stop drinking. However, an alternate assumption is that because she is having uncontrolled seizures and is driving, she presents an immediate risk to herself and others. Clinical Reasoning: This question requires clinical reasoning because it raises competing considerations for the immediate management of this patient with complex medical and psychosocial issues. The pharmacokinetics of phenobarbital, which is likely impacted by her alcohol consumption, have to be considered, as well as her recent change in employment and related lifestyle factors. Given that phenobarbital is a central nervous system depressant, it can interact with alcohol and exacerbate CNS depression which could be leading to the increased seizures. Moreover, alcohol can potentially be contributing to inefficacy of phenobarbital leading to uncontrolled seizures. However, the fact that she's driving on a regular basis with uncontrolled seizures presents an immediate and critical risk to her and public's safety. Concluding: After analyzing the available data, we learn that the immediate and acute risk of harm lies in the fact that she's driving with uncontrolled seizures. While it is true that alcohol might be potentiating her seizures, the direct threat at this moment is her risk of having a seizure while driving, which could potentially lead to a catastrophic outcome. Response: Thus, the answer to the question should be (D) Instructing the patient to stop driving. This course of action addresses the immediate risk of harm to both the patient and the public, thus making it the priority at this visit. However, as part of a comprehensive, long-term management plan, it is necessary to further investigate the patient's alcohol use as well (option C) and optimize her seizure disorder treatment regimen (option A). Evaluating the patient for depressive disorder (option B) might also be worth considering given her job loss and increased alcohol consumption. Nonetheless, instructing the patient to stop driving is the most immediate concern.

**AI_E:** RESTATING THE QUESTION: The given clinical case describes a woman who has a history of seizures, hypertension, and alcohol misuse, with increased seizures coinciding with loss of her job and increased alcohol intake. Given the range of risks and medical issues, we are asked to prioritize her management. She is currently driving, which poses a safety risk should she have a seizure while at the wheel. We have to choose among management approaches - changing treatment for seizures, assessing her for depression, dealing with her alcohol misuse, or addressing her unsafe driving. PLANNING: We can make several assumptions. One, the patient's increased alcohol intake may be affecting her medication management and thus her seizure control. Two, the stress of losing her job and the resulting depression could also impact her seizure control. Three, the uncontrolled seizures pose an immediate safety risk given her role as a driver. Four, her borderline hypertension and family history of diabetes and hypertension could present a long-term health risk. Given these, I hypothesize that the immediate safety risk posed by uncontrolled seizures while driving needs to be clinically prioritized. CLINICAL REASONING: In this case, the need to prevent potential injury or death due to a seizure while driving is immediate and clear. The alcohol misuse, while significant, may require a more prolonged approach and can be addressed in subsequent visits, possibly in conjunction with other supportive resources. Similarly, the uncontrolled seizures and potential depressive disorder will need to be managed, but these processes usually take time before a change is evident. Hypertension control is also important, but it doesn't have the same immediate and potential catastrophic impact as driving under risk of seizures. The immediate safety of the patient and others on the road should be the priority, requiring advising her to stop driving until her seizures are under control. CONCLUDING: Based on the clinical reasoning process, the immediate health risk to the patient and public safety due to potential for seizures while driving necessitates advising her to stop driving until her seizures are better controlled. Other health issues - such as the alcohol misuse, the control of her seizures, potential depressive disorder, and management of her hypertension - although very important, do not constitute such a pressing, life-threatening condition and can be addressed better once the immediate risk has been removed. RESPONSE: Therefore, the priority in management at today's visit should be: (D) Instructing the patient to stop driving.

**AI_A:** (1) RESTATING THE QUESTION: This question asks for the priority issue to be addressed during the current visit of a 45-year-old woman with a seizure disorder. Her seizures have increased in frequency recently despite her compliance with medication. She has also started drinking two beers daily after she lost her job three weeks ago, a period corresponding to the increase in seizures. Her medication includes phenobarbital, potentially interacting with her alcohol use. She has taken on driving duties after losing her job, which raises safety concerns considering her escalated seizures. (2) PLANNING: We should consider the assumption that her synthesized seizure medication with alcohol has created a situation where her seizures have increased in frequency. Alternatively, we can assume her alcoholism impairs her ability to absorb the medication properly. Another assumption could be that the increased seizures have a different cause altogether. However, based on a higher incidence of seizures after alcohol use and the known interaction between phenobarbital and alcohol, we should clinically prioritize exploring the impact of alcohol use on her seizures and medication effectiveness. (3) CLINICAL REASONING: Phenobarbital is a sedative that is used to control seizures. Its pharmacodynamics can be influenced by the concurrent intake of alcohol, as both can have a compounded depressant effect on the central nervous system. Furthermore, chronic alcohol usage can alter drug metabolism, leading to decreased therapeutic efficacy. In this case, the temporal correlation between the uptick in the patient's alcohol consumption and the increased frequency of her seizures implicates a possible drug-alcohol interaction. This may be enhancing the central nervous system depression, accelerating the metabolism of phenobarbital, or synergistically lowering the seizure threshold. We must also consider the immediate safety implications of the increase in seizures, especially now that the patient has taken up driving. (4) CONCLUDING: Given the evident safety risks posed by the uncontrolled seizures in the context of the patient's new driving responsibilities, we need an immediate solution. Although addressing the alcohol issue is important for the long-term effectiveness of the treatment and the general health of the patient, the immediate need is to curb the danger posed by potential seizures while driving. (5) RESPONSE: Therefore, the current priority should be (D) Instructing the patient to stop driving. While her need for income is understood, her safety, as well as that of her passengers and others on the road, is of the utmost importance. Subsequently, her increasing alcohol use and its impact on her seizure disorder would be dealt with to prevent further health complications.

**Facilitator** The consensus among the AI responses is that the immediate priority in managing the 45-year-old woman with a history of seizure disorder, who has been experiencing daily seizures for the past three weeks despite medication, is to instruct her to stop driving (D). This is due to the immediate and significant risk posed to both her and public safety, given the potential for a seizure while driving. The increased frequency of her seizures coincides with her job loss and increased alcohol consumption, suggesting a possible interaction between her medication (phenobarbital) and alcohol. However, while addressing her alcohol use and optimizing her seizure disorder treatment are important for her long-term health and should be part of a comprehensive management plan, these issues do not present the same immediate, life-threatening risk as her driving under the risk of seizures. Evaluating her for depressive disorder might also be worth considering given her job loss and increased alcohol consumption. Nonetheless, the immediate concern is to manage the acute risk posed by her uncontrolled seizures while driving.
